# Native Colombian Fruits and Their by-Products: Phenolic Profile, Antioxidant Activity and Hypoglycaemic Potential

**DOI:** 10.3390/foods8030089

**Published:** 2019-03-03

**Authors:** Monica Rosa Loizzo, Paolo Lucci, Oscar Núñez, Rosa Tundis, Michele Balzano, Natale Giuseppe Frega, Lanfranco Conte, Sabrina Moret, Daria Filatova, Encarnación Moyano, Deborah Pacetti

**Affiliations:** 1 Department of Pharmacy, Health and Nutritional Sciences, University of Calabria, 87036 Rende (CS), Italy; monica_rosa.loizzo@unical.it (M.R.L.); rosa.tundis@unical.it (R.T.); 2 Department of Agri-Food, Animal and Environmental Sciences, University of Udine, via Sondrio 2/a, 33100 Udine, Italy; lanfranco.conte@uniud.it (L.C.); sabrina.moret@uniud.it (S.M.); 3 Department of Chemical Engineering and Analytical Chemistry, University of Barcelona, Martí i Franquès 1-11, 08028 Barcelona, Spain; oscar.nunez@ub.edu (O.N.); daria.filatova@gmail.com (D.F.); encarna.moyano@ub.edu (E.M.); 4 Department of Agricultural, Food, and Environmental Sciences, Marche Polytechnic University, Via Brecce Bianche, 60131 Ancona, Italy; m.balzano@staff.univpm.it (M.B.); n.g.frega@univpm.it (N.G.F.); d.pacetti@staff.univpm.it (D.P.)

**Keywords:** Passifloraceae, Solanaceae, liquid chromatography, hypoglycaemic, α-amylase

## Abstract

The phenols and fatty acids profile and in vitro antioxidant and hypoglycaemic activity of seed, peel, pulp or pulp plus seeds of Colombian fruits from Solanaceae and Passifloraceae families were investigated. Ultra-High Performance Liquid Chromatography (UHPLC)-High Resolution Mass Spectrometry (HRMS) revealed the presence of chlorogenic acid as dominant phenolic compound in Solanaceae samples. Based on the Relative Antioxidant Score (RACI) and Global Antioxidant Score (GAS) values, *Solanum quitoense* peel showed the highest antioxidant potential among Solanaceae samples while *Passiflora tripartita* fruits exhibited the highest antioxidant effects among Passifloraceae samples. *P. ligularis* seeds were the most active as hypoglycaemic agent with IC_50_ values of 22.6 and 24.8 μg/mL against α-amylase and α-glucosidase, respectively. Considering that some of the most promising results were obtained by the processing waste portion, its use as functional ingredients should be considered for the development of nutraceutical products intended for patients with disturbance of glucose metabolism.

## 1. Introduction

Colombia is a country with high levels of biodiversity and is home to a wide variety of exotic fruits. Traditionally, tropical fruit are consumed locally; nowadays, increasing production and more efficient transportation and refrigeration systems have led to increased global consumption with considerable quantities of tropical fruits that are now exported on a global scale. Tropical fruits not only present an attractive and characteristic exotic taste and aroma, but also represent a valuable source of bioactive compounds beneficial for humans. However, given the diversity of Colombian native and exotic species, the health properties and the chemical composition of some of these fruits have not been sufficiently studied.

It is well known that fruit phytochemicals play an important role against many ailments, including heart disease, diabetes, high blood pressure, cancer, etc. Among them, diabetes mellitus (DM) is projected to reach pandemic proportion in the next 25 years [1]. The persistent hyperglycaemia status that characterises both forms of DM causes an increase in the production of reactive oxygen species (ROS) both of cytosolic and mitochondrial origin. ROS could determine the long-term deterioration of pancreatic islet β-cell by affecting mitochondrial Adenosine triphosphate (ATP) production that is necessary for insulin secretion. The consequent mitochondrial dysfunction influences insulin sensitivity within muscle, liver and adipose tissue [2]. One of the most common approaches to reduce the intake of sugar is by the reduction of their intestinal absorption using carbohydrates-hydrolysing enzymes inhibitors. In our previous research articles, the potential role of natural products as α-amylase and α-glucosidase inhibitors and antioxidant compounds was demonstrated [3,4,5].

In this context, to enhance scientific knowledge for the composition and health benefits of Colombian fruits and their by-products, we selected *Solanum quitoense*, *Physalis peruviana*, and *Cyphomandra betacea* (synonymous *Solanum betaceum* Cav) belonging to Solanaceae family, and *Passiflora pinnatistipula*, *Passiflora tripartita* and *Passiflora ligularis* belonging to Passifloraceae family.

*S. quitoense* has a characteristic *Citrus* flavour, sometimes described as a combination of rhubarb and lime [6]. Fruits are used to prepare juice or a drink called lulada. The fruit of *P. peruviana* is a smooth berry largely used to prepare snacks, pies or jams. It is used also in salads combined with avocado [7]. *Cyphomandra betacea* is one of the most popular fruit in South America where it is used to make juice. Fruits of *P. tripartita* are generally consumed raw or as ingredient of ice creams, fruit salads, pies, jellies or to make drinks [7]. *P. ligularis* fruit is also employed for the preparation of processed products like marmalade and jelly [3], while *P. pinnastipula* is consumed as drinks, ice-cream or marmalades [7].

After the selection, we have screened the fatty acids and the phenolics profile, as well as the antioxidant and hypoglycaemic potential of extracts from peel, pulp, seed or pulp plus seed of selected Colombian fruits in order to investigate the functional potential of each fruits portion, and to identify a potential use of the processing waste of these extracts as functional ingredients.

## 2. Materials and Methods

### 2.1. Chemicals and Reagents

All chemicals and reagents used in this study were purchased from Sigma-Aldrich Chemical Co. Ltd. (Milan, Italy) and VWR International (Milan, Italy) and, unless specified otherwise, were analytical grade or higher.

### 2.2. Plant Material and Extraction Procedure

Fruits were purchased at the local market in Bogotá (Colombia). At least 10 fruits were combined for each of the three replicated samples (one per month). Samples were examined and cleaned by using distilled water. Pulp, peel and seed were manually separated wherever it is possible in order to submit each portion to extraction procedure. The freeze-dried vegetable material (2.5 g of clean seeds or 5 g of peel or 5 g of pulp or seed + pulp) were finely ground with an Ultraturrax device (IKA^®^-Werke GmbH & Co. KG, Königswinter, Germany) and undergone to cold extraction with absolute ethanol (40 mL) for 24 h at room temperature (20 °C). The top phase was filtered through Whatman filter paper #4 (9.0 cm diameter) and the residue was re-extracted with 30 mL of absolute ethanol. The filtered top phases were combined and dried in a rotary evaporator (Buchi R-300, Milan, Italy) with operating temperature of 35 °C. Extraction yield ranged from 7.2 to 36.5 g ethanolic extracts on 100 g dry product in Solanaceae family and from 2.2 to 49.1 g/100 g dry product in Passifloraceae family. Extracts were then stored at −20 °C until further analysis.

### 2.3. Ultra-High Performance Liquid Chromatography (UHPLC)-High Resolution Mass Spectrometry (HRMS). Conditions and Analysis of Phenolic Compounds in Colombian Fruits

UHPLC-HRMS analysis was carried out using an Accela UHPLC system (Thermo Fisher Scientific, San Jose, CA, USA) equipped with a quaternary pump. For the chromatographic separation, an Ascentis Express C18 (150 × 2.1 mm, 2.7 µm, Supelco) column was employed. The mobile phase was a mixture of solvent A (0.1% formic acid in water) and solvent B (0.1% formic acid in acetonitrile) and the flow rate was 300 µL/min. Gradient elution program was run as stated: from 0–1 min, 10% B; 1–20 min, linear gradient from 10 to 95% B; 20–23 min, isocratic step at 95% B; 23–25 min, back to initial conditions at 10% B, and 25–30 min, isocratic step at 10% B for column re-equilibration. The UHPLC system was coupled to a Q-Exactive quadrupole-Orbitrap (Thermo Fisher Scientific) mass spectrometer equipped with a heated electrospray probe ionization source (H-ESI II). HESI-II was operated in negative ionisation mode. Nitrogen was used as a sheath gas, sweep gas and auxiliary gas at flow rates of 60, 0 and 10 a.u. (arbitrary units), respectively. Heater temperature was set at 350 °C. Capillary temperature was set at 320 °C and electrospray voltage at −2.5 kV. The HRMS instrument was operated in full MS scan with a m/z range from 100 to 1500, and the mass resolution tuned into 70,000 full width half maximum (FWHM) at m/z 200, with an automatic gain control (AGC) target (the number of ions to fill C-Trap) of 5.0e5 with a maximum injection time (IT) of 200 ms. Selected analytes belonging to different phenolic classes, namely gallic acid, (+)-catechin hydrate, p-coumaric acid, chlorogenic acid, (−)-epicatechin, ferulic acid, homogentisic acid, polydatin, syringaldehyde, taxifolin, umbelliferon, sinapic acid, kaempferol and vanillic acid, were monitored.

### 2.4. Fatty Acids Profile

An aliquot (20 mg) of ethanol extract from peel, seed and pulp of each fruit underwent alkaline transmethylation [8]. The gas-chromatography (GC) analysis of fatty acid methyl esters (FAME) was carried out using GC-430 apparatus (Varian, Palo Alto, CA, USA) equipped with a Flame Ionization Detector (FID) and a CPSil88 fused silica capillary column (100 m, 0.25 mm internal diameter, film thickness 0.2 μm, Chrompack, Middelburg, Netherlands). The carrier gas was helium at a flow rate of 1.6 mL/min; the oven temperature program started from 160 °C, rose to 240 °C at a rate of 4 °C/min and then remained at 240 °C for 10 min. The injector temperature was 260 °C. The sample was injected into a split/splitless system. Peaks were identified by comparison with known standards.

### 2.5. ABTS and DPPH Radical Scavenging Assays

The radical scavenging potential was investigated by using two different spectrophotometric methods: 2,2′-azino-bis (3-ethylbenzothiazoline-6-sulphonic acid (ABTS) and 2,2-diphenyl-1-picrylhydrazyl (DPPH) assays [9]. The radicals (ABTS or DPPH) scavenging ability was calculated as follows:

scavenging activity = [(A_0_ − A)/A_0_] × 100

where A_0_ is the absorbance of the control reaction and A is the absorbance in the presence of the extract. Ascorbic acid was used as positive control.

### 2.6. β-Carotene Bleaching Test

The protection of extract on lipid peroxidation was measured as previously described [9]. Briefly, β-carotene solution was added to linoleic acid and 100% Tween 20. The absorbance of the samples, standard and control was measured at 470 nm against a blank at t = 0 and successively at 30 and 60 min. Propyl gallate was used as positive control.

### 2.7. FRAP (Ferric Reducing Ability Power) Assay

The FRAP assay was applied following the procedure previously described [7]. The FRAP value represents the ratio between the slope of the linear plot for reducing Fe^3+^-TPTZ reagent by different Colombian fruits extract compared to the slope of the plot for FeSO_4_. Butylated hydroxytoluene (BHT) was used as positive control.

### 2.8. Relative Antioxidant Capacity Index (RACI) Calculation

Relative antioxidant capacity index (RACI) is a statistical application to integrate the antioxidant capacity values generated from different in vitro methods [10]. Thus, data obtained from ABTS, DPPH, β-carotene bleaching tests and FRAP tests were used to calculate RACI value for samples. The standard score is calculated by using the following Equation:

(x − μ)/σ

where x is the raw data, μ is the mean and σ is the standard deviation.

### 2.9. Global Antioxidant Score (GAS)

For each sample, the average of T-scores was used to calculate the GAS value. T-score is calculated by the following Equation:

T-score = (X − min)/(max − min)

where min and max, respectively, represent the smallest and largest values of variable X among the investigated extract [10].

### 2.10. a-Amylase and a-Glucosidase Inhibitory Assays

The α-amylase inhibitory test was performed as previously described [11]. Concisely, α-amylase solution, starch solution and colorimetric reagent were prepared. Control and samples at different concentrations were added to starch solution and left to react with the enzyme at room temperature for 5 min. The generation of maltose was quantified at 540 nm by the reduction of 3,5-dinitrosalicylic acid to 3-amino-5-nitrosalicylic acid.

The α-glucosidase inhibition was measured as previously described [11]. Both control and samples (at concentrations in the range 0.01–1 mg/mL) were added to maltose solution and left to equilibrate at 37 °C. The reaction was started by adding the enzyme and left to incubate at 37 °C for 30 min. A perchloric acid solution was used to stop the reaction. The supernatant was collected and mixed with peroxidase/glucose oxidase and *o*-dianisidine and left to incubate for 30 min at 37 °C. The absorbance was measured at 500 nm. Acarbose was the positive control in both tests.

### 2.11. Statistical Analysis

The inhibitory concentration 50% (IC_50_) was calculated by non-linear with the use of Prism Graphpad Prism version 4.0 for Windows, GraphPad Software, San Diego, CA, USA. Differences within and between groups were evaluated by one-way ANOVA followed by a multicomparison Dunnett’s test (α = 0.05): **** *p* < 0.0001, *** *p* < 0.001, ** *p* < 0.05, compared with the positive controls.

The concentration-response curve was obtained by plotting the percentage of inhibition versus the concentrations.

## 3. Results and Discussion

### 3.1. UHPLC-ESI-HRMS Phenolic Profile

The phenolic profile of the ethanol extracts of Colombian fruits was outlined by UHPLC-ESI-HRMS analysis (Table 1). A total of 16 phenolic compounds were detected. Concerning the Solanaceae family, *C. betacea* showed the highest phenol concentrations. In all fruits, the peel extracts presented higher phenol amounts than those derived from pulp and seed. Despite a different content, chlorogenic acid resulted in the most abundant compound in all extracts. An appreciable amount of rutin hydrate was found in the peel extract from *S. quitoense*. This finding was in agreement with data reported by Gancel et al. [12], who found that the main chlorogenic acid and dihydrocaffeoyl spermidine were located in all parts of *S. quitoense*, whereas the flavonol glycosides were exclusively present in the peel. *trans*-Cinnamic acid was recorded only in the *C. betacea* pulp extract. Moreover, although at low concentrations, ferulic acid and sinapic acid were revealed exclusively in peel and pulp extracts from *C. betacea*. A different phenolic profile was reported in *C. betacea* pulp (yellow variety) by Mertz et al. [13]. Besides chlorogenic acids, they found glycoside ester of caffeic and ferulic acids. These differences could be related to the different solvent used for phenolic extraction. In fact, acetone was used as extraction solvent [13]. Markedly different phenolic composition occurred in extracts from fruits belonging to Passifloraceae family. *P. pinnatistipula* peel extract showed *trans*-cinnamic acid as main component, followed by sinapic and gallic acids. In pulp extract, only two compounds were identified such as polydatin and rutin hydrate. The most abundant compound identified in seed extract was (−)-epicatechin. Differently, the peel extract of *P. tripartita* showed ferulic acid and (+)-catechin as main compounds. (+)-Catechin was more abundant in pulp and seed extracts, respectively. Moreover, the pulp was characterised by a high content of sinapic acid (211.4 ppm). The other identified compounds were in the range 0.2–11.9 ppm. Regarding *P. ligularis* fruit, the peel extract was mainly formed by (+)-catechin, whereas the pulp+seed extract was characterised by low total phenolic amounts (<5 ppm) with polydatin and vanillin as the main compounds. Unfortunately, no data about phenolic profile in the literature were found for the investigated Passifloraceae fruits.

### 3.2. Fatty Acid Composition

The average fatty acid composition of extracts from peel, pulp and seed of the investigated fruits is reported in Table 2. The fatty acid composition of the extracts changed according to the family fruit and to the part of the fruit. Generally, all extracts were mainly formed by essential fatty acids, such as α-linoleic (C18:2 n-6) and α-linolenic acid (C18:3 n-3) acids. In detail, linoleic acid represented the most abundant fatty acid in all Passifloraceae fruit extracts, except in the pulp extract from the *P. tripartita* fruit. This extract did not contain monounsaturated fatty acids. It presented four fatty acids: palmitic (C16:0), stearic (C18:0), linoleic and linolenic acids. The latter was the most abundant.

Concerning the Solanaceae family, α-linolenic acid (C18:3 n-6) was exclusively found in extracts from *P. peruviana*. Moreover, all extracts from *S. quitoense* and *P. peruviana* presented α-linolenic acid as major fatty acid. Differently, peel and pulp extracts from *C. betacea* presented the oleic acid (C18:1 n-9) as the predominant fatty acid, whereas the major fatty acid in seed extract was α-linoleic acid. In a previously study, Ramakrishnan et al. [14] investigated seed oil of *C. betacea* and they found that the most abundant fatty acids were linoleic (70.47%) and oleic (14.93%) acids.

### 3.3. Antioxidant Activity

Different tests were applied to screen the antioxidant activity of Colombian fruits. A concentration-effects relationship was found for all tested extracts in all tests except for FRAP assay (Table 3). DPPH and ABTS test were used to screen the radical scavenging potential. Among the Solanaceae family, the *S. quitoense* peel showed the highest DPPH radical scavenging potential with an IC_50_ of 38.8 μg/mL followed by *C. betacea* seed (IC_50_ of 57.9 μg/mL). Higher IC_50_ values were found when an ABTS assay was used. A promising protection of lipid peroxidation was observed with *S. quitoense* pulp+seed (IC_50_ of 6.9 μg/mL) followed by *P. peruviana* peel (IC_50_ of 10.2 μg/mL).

*C. betacea* peel exhibited a FRAP value similar to those reported for the positive control BHT (63.9 versus 63.2 μM Fe(II)/g). The antioxidant potential of *C. betacea* non-edible portion in terms of radicals scavenging ability and ferric reducing agent was previously described [15]. Several works evidenced also the antioxidant potential of *P. peruviana* whole fruit [9].

Considering Passifloraceae family, *P. tripartita* extracts showed the most promising DPPH radical scavenging activity with IC_50_ values of 3.2, 3.8, and 3.9 μg/mL for seed, pulp and peel, respectively. Additionally, peels exhibited an interesting ABTS^+^· radical scavenging potential (IC_50_ value of 50.1 μg/mL) and the best protection of lipid peroxidation (IC_50_ value of 3.8 μg/mL). Moreover, *P. tripartita* showed the highest ferric reducing ability and in particular seed exhibited a 1.4-time higher FRAP value than those reported for BHT. Analysis of the chemical profile revealed a high content of sinapic acid, (−)-epicatechin and ferulic acid in the pulp, seed and peel, respectively.

To get a ranking of Colombian fruits antioxidant capacity, relative antioxidant capacity index (RACI) and Global Antioxidant Score (GAS) were calculated. Both statistical applications were generated from the perspective of statistics by integrating the antioxidant capacity values generated from different in vitro methods (Table 3). Based on RACI and GAS value among fruits derived from Solanaceae family, *S. quitoense* peel had the highest antioxidant potential, while *P. tripartita* pulp exhibited the highest antioxidant capacity among investigated Passifloraceae fruits. Pearson’s correlations coefficient evidenced a positive correlation between the content of identified phenol and FRAP value for both Solanaceae and Passifloraceae (*r* = 0.79 and 0.81, respectively). Phenolic compounds showed redox properties acting as reducing agents, hydrogen donators and singlet oxygen quenchers [16]. Selected Colombian fruits have been shown to be a good source of phenols that are known to possess a great antioxidant potential. In particular, these phytochemicals are able to act as free radical scavengers, metal chelator and to protect lipids from peroxidation. Moreover, the antioxidant activity exerted by phenols is the results of influence on cell signalling pathways and on gene expression. The antioxidant activity results from a complex interaction between different compounds in phytocomplex, which produce synergistic effect.

### 3.4. Carbohydrate Hydrolysing Enzymes Inhibition by Colombian Fruits Extracts

The inhibition of carbohydrates-hydrolysing enzymes α-amylase and α-glucosidase was investigated and results are herein reported. All investigated samples inhibited both enzymes in a concentration-dependent manner, however, generally, α-glucosidase showed to be more sensible (Table 3). An analysis of the data evidenced that *S. quitoense* peel extract had the best activity with IC_50_ values of 27.9 and 31.8 μg/mL for α-glucosidase and α-amylase, respectively. Additionally, a promising hypoglycaemic activity was observed with *P. peruviana* peel that showed IC_50_ values of 34.1 and 37.6 μg/mL against α-amylase and α-glucosidase, respectively. A lower IC_50_ value was found with *C. betacea* peel against α-glucosidase (IC_50_ of 32.9 μg/mL). Among Passiflorareae fruits, *P. ligularis* pulp + seed extract showed the highest activity with IC_50_ values of IC_50_ values of 22.6 and 24.8 μg/mL against α-amylase and α-glucosidase, respectively, followed by *P. pinnatistipula* (IC_50_ values of 46.4 and 37.7 μg/mL against α-amylase and α-glucosidase, respectively). Several samples showed a lower IC_50_ value than those reported for the largely prescribed drugs acarbose herein used as positive control.

Among the identified phytochemicals, flavonoids were mainly involved in the management of Type 2 Diabetes Mellitus (T2DM). These compounds were able to (a) inhibit carbohydrates-hydrolysing enzymes; (b) inhibit sodium-dependent glucose transporter 1 (SGLT1); (c) stimulate insulin secretion; (d) reduce hepatic glucose output; (e) enhance insulin-dependent glucose uptake. In particular, rutin, which was particularly abundant in *S. quitoense* peel extract, inhibited both -amylase and α-glucosidase with IC_50_ values of 0.043 and 0.037 μM, respectively [17]. A similar consideration could be done for chlorogenic acid that inhibited both α-amylase and α-glucosidase with IC_50_ values of 25 and 26.1 μM, respectively [18]. Different hypoglycaemic mechanisms were reported in the literature for this phytochemical. It stimulates glucose uptake in skeletal muscle and suppression of hepatic glucose production by the activation of AMPK. In addition, it has been found that it could modulate glucose in both genetically and healthy metabolic related disorders including DM [19]. Moreover, *S. quitoense* peel extract was rich in linoleic acid. This acid showed a more potent α-glucosidase inhibitory activity than acarbose, whereas a weaker anti α-amylase activity was observed [20].

## 4. Conclusions

In the present study, we investigated the phenols and fatty acids profile and in vitro antioxidant and hypoglycaemic activity of edible and non-edible parts of Colombian fruits from Solanaceae and Passifloraceae families. The phenolic profile of the ethanol extracts of selected exotic fruits was reported herein for the first time. A total of 16 phenolic compounds were detected, with peel extracts showing the highest amount of phenolics compared to pulp and seed samples. Generally, chlorogenic acid and rutin hydrate were the most representative compounds. Considering only the fruit species in which it was possible to separate peel, pulp and seed, the results showed how the omega-3 fatty acids preferentially accumulated in the pulp and in the peel rather than in the seeds. Furthermore, it was found that *S. quitoense*, which contains considerable amount of rutin hydrate, had the highest antioxidant potential among the Solanaceae samples, while *P. tripartita* fruits exhibited the highest antioxidant capacity among the investigated Passifloraceae samples. Moreover, carbohydrate-hydrolysing enzyme inhibitory activity studies highlighted a promising hypoglycaemic activity of *P. ligularis* seed and *S. quitoense* peel samples. The latter demonstrated that carbohydrates-hydrolysing enzymes inhibition was higher than the commercial drug acarbose. Although some identified flavonoids possess hypoglycaemic activity, it is not possible to attribute these interesting properties to these compounds, but rather to the phytocomplex in which a synergism of action can occur.

Overall, the present results supported the possibility of using peels or seeds, which are normally considered as food by-products of juice production, as potential sources of bioactive ingredients in functional beverages, nutraceutical or dietary supplement formulations for the treatment and/or prevention of several diseases associated with oxidative stress such as type 2 diabetes.

## Figures and Tables

**Table 1:** Quantification (mg/kg ethanol extract) of identified phenolic compounds in Colombian fruits from Solanaceae and Passifloraceae families.

	(+)-Catechin	Chlorogenic Acid	*trans*-Cinnamic Acid	*p*-Coumaric Acid	(−)-Epicatechin	Ferulic Acid	Gallic Acid	Homogentisic Acid	Polydatin	Rutin Hydrate	Sinapic Acid	Syringaldehyde	Taxifolin	Vanillic Acid	Vanillin	Veratric Acid	⅀Phenolic
**Solanaceae**																	
*S. quitoense*																	
Peel	n.d.	98.6 ± 2.9	n.d.	0.4 ± 0.0	n.d.	n.d.	1.3 ± 0.0	n.d.	n.d.	51.1 ± 3.6	n.d.	n.d.	0.3 ± 0.0	n.d.	n.d.	n.d.	151.6 ± 2.5
Pulp + Seeds	n.d.	19.1 ± 1.0	n.d.	n.d.	n.d.	n.d.	n.d.	n.d.	n.d.	n.d.	n.d.	n.d.	0.1 ± 0.0	n.d.	n.d.	n.d.	20.0 ± 1.0
*P. peruviana*																	
Peel	n.d.	1.5 ± 0.1	n.d.	n.d.	n.d.	n.d.	0.9 ± 0.0	n.d.	0.6 ± 0.03	0.1 ± 0.0	n.d.	n.d.	n.d.	n.d.	n.d.	n.d.	3.2 ± 0.2
Pulp + Seeds	n.d.	0.7 ± 0.1	n.d.	n.d.	n.d.	n.d.	n.d.	n.d.	0.6 ± 0.07	n.d.	n.d.	n.d.	n.d.	n.d.	n.d.	n.d.	1.4 ± 0.2
*C. betacea*																	
Peel	n.d.	253.8 ± 3.8	n.d.	0.2 ± 0.0	n.d.	8.7 ± 0.2	n.d.	n.d.	n.d.	9.5 ± 1.4	10.3 ± 0.7	0.7 ± 0.0	0.2 ± 0.0	n.d.	n.d.	n.d.	284.1 ± 1.2
Pulp	n.d.	125.5 ± 1.2	28.7 ± 2.0	0.2 ± 0.0	n.d.	7.6 ± 0.1	n.d.	n.d.	n.d.	n.d.	1.6 ± 0.2	n.d.	0.1 ± 0.0	n.d.	n.d.	n.d.	165.1 ± 2.2
Seeds	0.8 ± 0.2	37.7 ± 1.3	n.d.	n.d.	2.5 ± 0.1	n.d.	n.d.	n.d.	n.d.	0.7 ± 0.0	n.d.	n.d.	0.1 ± 0.0	n.d.	n.d.	n.d.	42.1 ± 1.2
**Passifloracea**																	
*P. pinnatistipula*																	
Peel	n.d.	n.d.	132.4 ± 5.1	1.5 ± 0.0	2.4 ± 0.2	4.6 ± 0.5	8.1 ± 0.2	n.d.	1.1 ± 0.03	4.7 ± 0.2	9.4 ± 0.1	n.d.	1.2 ± 0.0	n.d.	n.d.	n.d.	166.2 ± 3.2
Pulp	n.d.	n.d.	n.d.	n.d.	n.d.	n.d.	n.d.	n.d.	0.5 ± 0.02	0.4 ± 0.1	n.d.	n.d.	n.d.	n.d.	n.d.	n.d.	1.0 ± 0.1
Seeds	n.d.	n.d.	n.d.	0.3 ± 0.0	8.1 ± 0.8	n.d.	n.d.	n.d.	3.4 ± 0.22	n.d.	n.d.	n.d.	0.2 ± 0.0	n.d.	n.d.	n.d.	12.4 ± 0.3
*P. tripartita*																	
Peel	40.1 ± 2.0	n.d.	n.d.	2.0 ± 0.1	n.d.	47.8 ± 1.8	n.d.	n.d.	0.7 ± 0.05	n.d.	10.2 ± 0.9	n.d.	n.d.	0.5 ± 0.0	n.d.	n.d.	101.9 ± 1.3
Pulp	140.9 ± 8.2	n.d.	n.d.	1.8 ± 0.2	7.9 ± 0.5	16.3 ± 1.4	n.d.	n.d.	1.9 ± 0.27	n.d.	211.4 ± 1.5	0.8 ± 0.0	n.d.	1.3 ± 0.0	n.d.	n.d.	383.3 ± 1.3
Seeds	208.8 ± 3.6	n.d.	n.d.	n.d.	81.5 ± 3.5	n.d.	n.d.	n.d.	0.7 ± 0.03	n.d.	55.3 ± 4.1	2.2 ± 0.1	2.1 ± 0.1	n.d.	n.d.	42.6 ± 1.7	394.2 ± 2.6
*P. ligularis*																	
Peel	257.6 ± 4.2	0.2 ± 0.0	n.d.	1.6 ± 0.1	n.d.	11.9 ± 1.9	2.4 ± 0.3	8.95 ± 1.1	2.1 ± 0.08	n.d.	4.0 ± 0.2	2.6 ± 0.1	n.d.	n.d.	5.2 ± 0.1	n.d.	297.5 ± 1.4
Pulp + Seeds	n.d.	n.d.	n.d.	0.7 ± 0.1	n.d.	n.d.	n.d.	n.d.	1.7 ± 0.04	0.3 ± 0.0	n.d.	n.d.	n.d.	n.d.	1.8 ± 0.0	n.d.	4.4 ± 0.9

n.d.: not detectable (<LOD); Limit of detection (LOD): (+)-catechin: 10 µg/kg; chlorogenic acid: 30 µg/kg; *trans*-cinnamic acid: 20 µg/kg; *p*-coumaric acid: 18 µg/kg; (−)-epicatechin: 10 µg/kg; ferulic acid: 50 µg/kg; gallic acid: 27 µg/kg; homogentisic acid: 30 µg/kg; polydatin: 10 µg/kg; rutin: 10 µg/kg; sinapic acid: 30 µg/kg; syringinc acid: 50 µg/kg; taxifolin: 10 µg/kg; vanillic acid: 35 µg/kg; vanillin: 50 µg/kg; veratric acid: 50 µg/kg.

**Table 2:** Fatty acid (% of total fatty acids) composition of peel, pulp and seed of Colombian fruits from Solanaceae and Passifloraceae families.

Fatty Acids	*S. Quitoense*		*P. Peruviana*		*C. Betacea*			*P. Pinnatistipula*			*P. Tripartita*		*P. Ligularis*		
	Peel	Pulp + Seed	Peel	Pulp + Seed	Peel	Pulp	Seed	Peel	Pulp	Seed	Peel	Pulp	Seed	Peel	Pulp + Seed
C16:0	22.6 ± 0.1	25.6 ± 0.4	20.6 ± 2.6	19.5 ± 0.7	22.9 ± 0.4	19.8 ± 0.1	13.6 ± 0.0	15.4 ± 0.1	15.6 ± 0.1	11.8 ± 0.0	24.0 ± 0.2	19.1 ± 0.0	9.6 ± 0.0	34.0 ± 0.2	7.8 ± 0.0
C18:0	6.3 ± 0.2	4.7 ± 0.2	2.7 ± 0.1	2.4 ± 0.1	2.6 ± 0.2	1.4 ± 0.1	3.3 ± 0.1	2.2 ± 0.0	2.9 ± 0.1	1.9 ± 0.0	n.d.	25.0 ± 0.1	1.9 ± 0.0	2.1 ± 0.0	2.5 ± 0.0
C20:0	n.d.	n.d.	5.4 ± 0.5	n.d.	n.d.	n.d.	n.d.	n.d.	n.d.	n.d.	n.d.	n.d.	n.d.	n.d.	n.d.
SFA	28.9 ± 0.1	30.3 ± 0.6	28.8 ± 2.1	22.0 ± 0.5	25.4 ± 0.2	21.5 ± 0.2	16.9 ± 0.1	17.6 ± 0.1	18.5 ± 0.2	13.6 ± 0.0	24.0 ± 0.2	44.1 ± 0.1	11.5 ± 0.0	36.2 ± 0.2	10.3 ± 0.1
C16:1	1.6 ± 0.1	n.d.	0.6 ± 0.0	1.4 ± 0.1	n.d.	1.8 ± 0.1	0.7 ± 0.0	0.4 ± 0.0	4.7 ± 0.1	0.2 ± 0.0	n.d.	n.d.	n.d.	n.d.	0.2 ± 0.0
C18:1 9	12.7 ± 0.3	17.0 ± 0.1	26.5 ± 0.5	20.5 ± 0.5	41.4 ± 0.3	39.1 ± 0.2	17.2 ± 0.6	11.3 ± 0.0	7.5 ± 0.1	10.0 ± 0.0	5.7 ± 0.3	n.d.	10.8 ± 0.1	3.3 ± 0.0	15.1 ± 0.3
C18:1 11	3.6 ± 0.3	n.d.	6.4 ± 0.1	8.4 ± 0.4	2.3 ± 0.1	1.2 ± 0.1	1.4 ± 0.1	1.2 ± 0.0	8.1 ± 0.1	0.2 ± 0.0	n.d.	n.d.	n.d.	0.9 ± 0.1	0.3 ± 0.0
MUFA	18.0 ± 0.7	17.0 ± 0.1	33.5 ± 0.6	30.3 ± 1.0	43.7 ± 0.4	41.7 ± 0.4	19.3 ± 0.8	13.0 ± 0.1	20.3 ± 0.2	10.4 ± 0.0	5.7 ± 0.3	n.d.	10.8 ± 0.1	4.2 ± 0.1	15.6 ± 0.4
C18:2 n-6	11.5 ± 0.1	23.5 ± 0.9	3.6 ± 0.1	11.9 ± 0.1	10.4 ± 0.1	13.1 ± 0.2	58.3 ± 1.0	63.9 ± 0.3	41.0 ± 0.4	75.5 ± 1.4	39.9 ± 0.3	16.6 ± 0.0	76.6 ± 0.3	36.0 ± 0.1	73.3 ± 0.7
C18:3 n-6	n.d.	n.d.	4.3 ± 0.1	4.4 ± 0.7	n.d.	n.d.	n.d.	n.d.	n.d.	n.d.	n.d.	n.d.	n.d.	n.d.	n.d.
C18:3 n-3	41.6 ± 0.9	29.2 ± 0.5	29.8 ± 1.3	31.4 ± 1.0	20.5 ± 0.4	23.6 ± 0.7	5.5 ± 0.1	5.5 ± 0.0	20.2 ± 0.0	0.5 ± 0.0	30.4 ± 0.2	39.3 ± 0.1	1.0 ± 0.0	23.6 ± 0.0	0.8 ± 0.0
PUFA	53.1 ± 0.8	52.7 ± 0.5	37.7 ± 1.5	47.7 ± 0.4	30.9 ± 0.3	36.8 ± 0.5	63.8 ± 0.9	69.4 ± 0.2	61.2 ± 0.4	76.0 ± 0.0	70.4 ± 0.1	55.9 ± 0.1	77.7 ± 0.1	59.6 ± 0.1	74.1 ± 1.4

SFA, saturated fatty acid; MUFA, monounsaturated fatty acid; PUFA, polyunsaturated fatty acid; n.d.; not detectable. Results represents means ± standard deviation (S.D.) (*n* = 3).

**Table 3:** In vitro antioxidant and hypoglycaemic activity of selected Colombian fruits belonging to Solanaceae and Passifloraceae family.

Title	Sample	DPPH (IC_50_ μg/mL)	ABTS (IC_50_ μg/mL)	β-Carotene Bleacing Test (IC_50_ μg/mL)	FRAP (μM Fe(II)/g)	RACI	GAS	α-Amylase (IC_50_ μg/mL)	α-Glucosidase (IC_50_ μg/mL)
Solanaceae									
*S. quitoense*	Peel	38.8 ± 2.1 ****	167.6 ± 3.7 ****	11.1 ± 1.3 ****	49.4 ± 1.5 ****	−0.43	0.81	31.8 ± 1.2 ****	27.9 ± 1.0
	Pulp + Seed	61.3 ± 2.0 ****	576.8 ± 7.5 ****	6.9 ± 0.5	16.2 ± 2.5 ****	−0.38	0.87	54.9 ± 2.4	57.1 ± 2.0 ****
*P. peruviana*	Peel	117.9 ± 5.1 ****	843.3 ± 3.9 ****	10.2 ± 1.1 ***	13.0 ± 0.8 ****	0.20	1.72	34.1 ± 2.2 ****	37.6 ± 2.5
	Pulp + Seed	65.3 ± 2.1 ****	> 1000	19.3 ± 1.6 ****	7.3 ± 0.6 ****	−0.03	1.40	64.3 ± 3.0 ****	45.0 ± 1.1
*C. betacea*	Peel	74.7 ± 3.5 ****	149.8 ± 3.6 ****	21.9 ± 1.5 ****	63.9 ± 3.5	0.05	1.52	77.1 ± 3.3 ****	32.9 ± 2.9 **
	Pulp	141.3 ± 5.5 ****	463.8 ± 4.7 ****	93.8 ± 4.5 ****	9.8 ± 0.6 ****	0.68	2.42	92.7 ± 3.8 ****	95.1 ± 4.6 ****
	Seed	57.9 ± 1.5 ****	329.8 ± 3.3 ****	58.2 ± 4.0 ****	25.4 ± 2.5 ****	−0.09	1.30	102.9 ± 4.0 ****	195.1 ± 4.7 ****
Passifloraceae									
*P. pinnatistipula*	Peel	207.9 ± 2.5 ****	125.3 ± 1.5 ****	>1000	40.5 ± 1.4 ****	0.23	1.47	46.4 ± 2.2	37.7 ± 2.2
	Pulp	671.9 ± 5.7 ****	151.7 ± 1.7 ****	50.6 ± 2.5 ****	3.2 ± 0.7 ****	0.11	1.15	78.2 ± 3.7 ****	44.1 ± 2.3
	Seed	372.2 ± 4.0 ****	>1000	133.8 ± 2.7 ****	28.7 ± 2.3 ****	0.59	1.74	54.1 ± 2.5	40.6 ± 2.8
*P. tripartita*	Peel	3.9 ± 0.8	177.8 ± 3.2 ****	>1000	22.7 ± 2.2 ****	0.03	1.18	86.3 ± 3.0 ****	56.1 ± 3.1 ****
	Pulp	3.8 ± 0.5	50.1 ± 2.5 ****	3.8 ± 0.3 ****	64.1 ± 3.4	−0.59	0.62	67.5 ± 3.7 ****	78.4 ± 3.9 ****
	Seed	3.2 ± 0.2	96.2 ± 3.7 ****	14.8 ± 0.7 ****	92.0 ± 3.7 ****	−0.49	0.27	52.7 ± 3.5	54.6 ± 3.2 ****
*P. ligularis*	Peel	61.3 ± 2.2 ****	282.1 ± 4.0 ****	265.1 ± 4.0 ****	39.9 ± 4.3 ****	−0.22	0.69	122.7 ± 4.5 ****	154.9 ± 3.9 ****
	Pulp + Seed	73.9 ± 2.7 ****	223.9 ± 3.7 ****	116.0 ± 3.1 ****	42.9 ± 3.8 ****	0.35	1.40	22.6 ± 3.7 ****	24.8 ± 3.9 **
Positive controls	Propyl gallate ^a^			1.0 ± 0.01					
	Ascorbic acid ^a^	2.0 ± 0.01	1.7 ± 0.8						
	BHT ^a^				63.2 ± 4.5				
	Acarbose ^a^							50.0 ± 0.9	35.5 ± 1.2

Data are given as media ± S.D. (*n* = 3); 2,2-diphenyl-1-picrylhydrazyl (DPPH) Radical Scavenging Activity Assay; Antioxidant Capacity Determined by Radical Cation 2,2′-Azino-bis(3-ethylbenzothiazoline-6-sulfonic acid) (ABTS^+^), β-Carotene bleaching test, Ferric Reducing Ability Power (FRAP); Relative antioxidant capacity Index (RACI); Global Antioxidant Score (GAS); ^a^ Propyl gallate, ascorbic acid and butylated hydroxytoluene (BHT) were used as positive control in antioxidant test, while acarbose was used in carbohydrate hydrolysing enzyme inhibition assays. Differences within and between groups were evaluated by one-way ANOVA followed by a multicomparison Dunnett’s test (=0.05): **** *p* < 0.0001, *** *p* < 0.001, ** *p* < 0.05 compared with the positive controls.
